# Adding Pioglitazone to Insulin Containing Regimens in Type 2 Diabetes: Systematic Review and Meta-Analysis

**DOI:** 10.1371/journal.pone.0006112

**Published:** 2009-07-01

**Authors:** Christine Clar, Pamela Royle, Norman Waugh

**Affiliations:** Department of Public Health, University of Aberdeen, Foresterhill, Aberdeen, United Kingdom; Johns Hopkins Bloomberg School of Public Health, United States of America

## Abstract

**Background:**

Type 2 diabetes is treated in a stepwise manner, progressing from diet and physical activity to oral antidiabetic agents and insulin. The oral agent pioglitazone is licensed for use with insulin when metformin is contraindicated or not tolerated. This systematic review and meta-analysis investigates the extent to which adding pioglitazone to insulin-containing regimens produces benefits in terms of patient-relevant outcomes.

**Methodology/Principal Findings:**

Medline, Embase, and the Cochrane Library were searched for randomised controlled trials comparing pioglitazone in combination with any insulin-containing regimen in comparison with the same insulin regimen alone in patients with type 2 diabetes. Outcomes investigated included HbA1c, hypoglycaemia, weight, and adverse events. Studies were selected, assessed and summarised according to standard systematic review methodology and in a meta-analysis. We included eight trials that examined the benefits of adding pioglitazone to an insulin regimen and studied a total of 3092 patients with type 2 diabetes. All studies included patients with previously inadequate glucose control. Trial duration was between 12 weeks and 34.5 months. The trials used pioglitazone doses of up to 45 mg/day. In our meta-analysis, the mean reduction in HbA1c was 0.58% (95% CI: −0.70, −0.46, p<0.00001). Hypoglycaemic episodes were slightly more frequent in the pioglitazone arms (relative risk 1.27; 95% CI: 0.99, 1.63, p = 0.06). Where reported, HDL-cholesterol tended to be increased with pioglitazone. Patients on pioglitazone tended to gain more weight than those who were not, with an average difference of almost 3 kg. Peripheral oedema was more frequent in the pioglitazone groups. None of the studies reported on fractures in women, and data on cardiovascular events were inconclusive, with most studies being too short or too small to assess these long-term outcomes.

**Conclusions/Significance:**

When added to insulin regimens, pioglitazone confers a small advantage in terms of HbA1c in type 2 diabetes patients with previous inadequate glucose control, but at the cost of increased hypoglycaemia and weight gain. Other considerations include the risk of heart failure, fractures in women, a reduced insulin dose, and the net financial cost.

## Introduction

Type 2 diabetes is usually seen in people who are overweight or obese, particularly if inactive. They usually have insulin resistance, and therefore require higher levels of insulin in order to keep blood glucose within the normal range. The pancreatic beta cell is initially able to compensate for insulin resistance by increasing production, thereby maintaining normal blood glucose levels. However, in most patients, pancreatic beta cell function progressively declines, leading to hyperglycaemia and clinical diabetes[1]. In the United Kingdom Prospective Diabetes Study (UKPDS), beta-cell function was found to be impaired at diagnosis, especially in patients who were not overweight[2].

The difficulty in maintaining metabolic control over time may be related to several behavioural factors (for example difficulties with healthy eating, exercise, medication regimens) but also reflects a progressive decline in beta-cell function[3], [4].

Type 2 diabetes has traditionally been treated in a stepwise manner, starting with lifestyle modifications and encouragement of physical activity and when necessary, pharmacotherapy with oral agents (NICE guideline)[5]. If control remains inadequate, insulin may be used, with or without combination with one or more oral agents. There is no clear consensus on the definition of “inadequate control”, but a consensus statement (2009) of a working group drawn from the American Diabetes Association and the European Association for the Study of Diabetes suggested that “an HbA1c over 7% should serve as a call to action to initiate or change therapy”[6].

Several classes of oral agents are available. These include the insulin secretagogues which stimulate the pancreas to release more insulin, by binding to a sulphonylurea receptor, the main group being the sulphonylureas; a second class are the insulin sensitizers, including the biguanide metformin and the thiazolidinediones rosiglitazone and pioglitazone; thirdly there are drugs that delay the absorption of carbohydrates from the gastrointestinal tract, such as acarbose; fourthly there are the DPP-IV inhibitors (also known as the gliptins), which extend the life of endogenous glucagon-like peptide. These include sitagliptin and vildagliptin (with more in development).

### The glitazones

The thiazolidinediones – or glitazones for short – decrease insulin resistance in muscle and adipose tissue by activating the peroxisome proliferator-activated receptor-gamma (PPAR- gamma) which increases production of proteins involved in glucose uptake. They also decrease hepatic glucose production by improving hepatic insulin sensitivity.

According to the Prescribing Support Unit (PSU), in collaboration with the York and Humber Public Health Observatory (YHPHO)[7], the glitazones are the third most used diabetes drugs in England (about 2.4 million prescriptions a year), after metformin (about 10 million prescriptions a year), and the sulphonylureas (around 5 million prescriptions a year). In terms of cost per annum, the glitazones are by far the most costly, being recently introduced drugs with no generic forms.

In addition to being used alone or in combination with other oral agents, pioglitazone is also licensed (EMEA 2008)[8] for use in combination with insulin in type 2 diabetes patients with insufficient glycaemic control on insulin, and for whom metformin is inappropriate because of contraindications or intolerance. In this review, we concentrate on this indication.

A Cochrane review of pioglitazone therapy in general by Richter et al. (2006)[9] included 22 trials which randomised a total of 6200 people to pioglitazone treatment. Most studies were of short duration. Published studies of at least 24 weeks pioglitazone treatment in people with type 2 diabetes mellitus did not provide convincing evidence of benefit in patient oriented outcomes like mortality, morbidity, adverse effects, costs and health-related quality of life. Metabolic control measured by HbA1c did not demonstrate clinically relevant differences to other oral glucose lowering drugs. The occurrence of oedema was significantly raised.

The only exception to the short-term trials found in the Cochrane review was the PROactive study[10]. This placebo controlled randomised trial of 5238 patients set out to determine the effect of pioglitazone on macrovascular morbidity and mortality in patients with type 2 diabetes who had evidence of macrovascular disease. Patients continued their other diabetes medications, mainly metformin, sulphonylureas, insulin, or combinations thereof. The primary end-point was a composite of death and non-fatal cardiovascular outcomes. The average time of observation was 34.5 months. The pioglitazone group had a lower risk but this did not reach statistical significance (HR 0.90, 95% CI 0.80 to 1.02; p = 0.095) despite the large numbers of recruits and events (at least one end-point event in 514 of the pioglitazone group and 572 of the placebo group). A secondary endpoint measure of death, non-fatal MI and stroke did reach statistical significance: HR 0.84, 0.72–0.98; p = 0.027.

However, oedema and heart failure were commoner in the pioglitazone group, with 11% reported as having heart failure compared to 8% in the placebo group; the proportions needing hospital admission were 6% and 4%. The death rates from heart failure showed no difference. Heart failure was not defined centrally, but was “as judged by the investigator”. Another outcome was “oedema in the absence of heart failure”. Heart failure can be difficult to diagnose, and the absence of any difference in mortality from heart disease, might suggest that it could have been over-diagnosed. However, an independent group of cardiologists reviewed all the cases of serious heart failure and concluded that it did occur more frequently in the pioglitazone group (5.5% versus 4.2% for placebo)[11].

Another finding from PROactive was that progression to needing insulin was halved in the pioglitazone group. At the start of the study, about one-third of the patients were on insulin. Their mean age was 62, mean BMI 31, and duration of diabetes 8 years; 75% had a history of hypertension and mean HbA1c was around 7.8%. The protocol asked investigators to aim for an HbA1c of <6.5%. By the end of follow-up, 11% of the pioglitazone group and 21% of the placebo group were on insulin treatment. The switch to insulin started early in the trial, presumably due to investigators trying to achieve the HbA1c target.

As concerns evidence on a combination of insulin and a glitazone, Strowig and Raskin (2005)[12] carried out a review of combination therapy with insulin and either metformin or a glitazone, or both. Details of methods are not given and the review was probably not systematic. The authors concluded that it was worthwhile continuing an insulin sensitiser in type 2 diabetes patients switched to insulin. Because metformin and glitazones have different balances of sites of preferential action (acting on glucose production and glucose disposal), they also made the case that triple therapy may be considered. Bailey (2005)[13] also supported combination therapy with metformin and a glitazone for reducing insulin resistance in type 2 diabetes.

### Objectives

This review investigates the extent to which adding pioglitazone to insulin-containing regimens affects glycaemic control, hypoglycaemia, weight change, lipids, and adverse events.

## Methods

### Study characteristics

We considered randomised controlled trials of pioglitazone in combination with any insulin regimen in patients of any age and gender with type 2 diabetes. Minimum trial duration was 12 weeks. Pioglitazone in combination with any insulin regimen (long-acting, twice daily mixture, both with or without additional oral medication (generally metformin and/or sulphonylurea)) was compared to the same insulin regimen (with or without the same additional oral regimen) given on its own. As outcome measures, we considered HbA1c, frequency of hypoglycaemia (especially if severe), glycaemic excursions (including post-prandial hyperglycaemia), total daily dose of insulin, weight change, changes in cardiovascular risk factors, and other adverse events.

We used a group of outcomes because the decision to use pioglitazone was expected to depend on trade-offs amongst them, such as better control versus weight gain. We would have considered diabetic secondary complication rates (retinopathy, nephropathy, myocardial infarction, angina, heart failure, stroke, amputation, death), and health-related quality of life, but it rapidly became clear that data would not be available.

### Ethics

As this was a systematic review of published literature, ethics approval was not required.

### Searching

Medline, the Cochrane Library, and Embase were searched for studies published between 1996 and 2008. The following Medline search strategy (Ovid) was adapted for use with the other databases:

1. exp Thiazolidinediones/
2. pioglitazone.tw.
3. 1 or 2
4. randomized controlled trial.pt.
5. meta-analysis.pt.
6. (random$ or meta-analysis or systematic review).tw.
7. 4 or 5 or 6
8. 3 and 7

In addition, reference lists of retrieved studies were checked.

Searches were also done to identify emerging evidence, from conference abstracts and trial registers.

### Selection

Studies were selected independently by two authors (PR and NW) based on the inclusion criteria listed above. Any discrepancies were resolved by discussion.

### Validity assessment

Randomised controlled trials were assessed on the following criteria based on the NICE guidelines manual[14]: Method of randomisation, allocation concealment, blinding of participants and outcome assessors, intention-to-treat analysis, proportion of participants excluded/lost to follow-up, power calculation, comparability of groups at baseline. Subgroup analysis based on quality was carried out for five or six or more quality criteria met versus fewer than five or six quality criteria met (i.e. two analyses with different cut-offs were done).

### Data abstraction

Data extraction was carried out by one researcher and checked by another. Any disagreements were resolved through discussion, involving a third person if necessary.

### Data synthesis

The clinical effectiveness, relative to the key comparators, was assessed, in terms of difference in effect size.

Data were summarised in a meta-analysis and using tables and text. For dichotomous outcomes, risk ratios were calculated and a Mantel-Haenszel random effects model was used. For continuous outcomes, weighted mean differences were calculated and an inverse variance random effects model was used. Where not directly available, standard deviations required in the meta-analysis were converted from standard errors; if a measure of variability was not given and standard deviations were available for at least 50% of included studies, the mean of the standard deviations of the remaining studies was used. Heterogeneity was assessed using the chi-squared test.

## Results

### Trial flow

**Figure 1:**
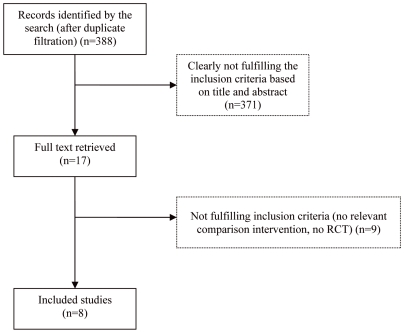
Flow chart of search results.

Eleven papers were identified as potentially relevant randomised controlled trials. Of these, eight fulfilled the inclusion criteria and compared pioglitazone plus insulin with insulin [15]–[22]. The remaining trials were excluded because they did not examine the comparison of interest and one was the uncontrolled extension of a trial that seemed relevant but could not be identified (see Figure 1).

### Study characteristics

Characteristics of the included trials are shown in Table 1.

**Table 1:** Characteristics of randomised placebo controlled trials included in the systematic review.

Study and Country	Focus	Interventions	Characteristics of Participants	Study Duration	Outcomes measured
*Asnani 2006* USA[15]	effect of pioglitazone on vascular reactivity in patients with insulin-treated type 2 diabetes	**1) PIO + ins:** pioglitazone 30 mg at breakfast, insulin continued as before **2) P + ins:** placebo, insulin continued as before **co-interventions:** stable lipid-lowering (statins) and antihypertensive therapy	**number:** 20 (10/10) **mean age:** 59/57 yrs **gender:** NR **BMI:** NR **ethnicity:** NR **diabetes duration:** 17/11 years **previous medication:** NR	4 months	**primary:** flow-mediated dilatation **other:** HbA1c, brachial artery reactivity, laboratory assessments, lipid profile
*Berhanu 2007* USA[16]	safety and efficacy of pioglitazone alone or in combination with metformin in reducing insulin dosage requirements for improved glycaemic control in patients with type 2 diabetes	**1) PIO + ins:** pioglitazone titrated to 45 mg/day during first 4 weeks of treatment, plus insulin as below **2) P + ins:** identical placebo plus insulin as below **both:** daily injections of Humalog, Humulin 70/30 or Humulin N **co-interventions:** maintained stable metformin and, as applicable, previous statin use	**number:** 222 (110/112) **mean age:** 52.9/52.5 yrs **gender (% female):** 56.4/58.9 **BMI:** 30.7/31.8 kg/m^2^ **Ethnicity(%):** Hispanic 50.0/58.9, non-Hispanic white 34.9/25.9, non-Hispanic black 12.7/11.6, other 2.7/3.6 **diabetes duration:** 7.7/8.5 yrs **previous medication (%):** SU+ MET: 90.0/92.9, insulin and MET: 8.2/5.4, insulin only:1.8/1.8	20 weeks	**primary:** change in insulin dosage from baseline to study end **other:** HbA1c, hypoglycaemia, total daily dose, weight change, adverse event, lipid parameters, C-peptide
*Fernandez 2008* USA[17]	relationship between glycaemic control, vascular reactivity and inflammation in type 2 diabetes	**1) PIO + ins:** pioglitazone 45 mg/day; started at 15 mg daily, 30 mg daily in week 2, 45 mg daily in week 4 **2) P + ins:** placebo (the ramipril + ins arm not considered here) **both:** patients selected between basal-bolus therapy (bedtime insulin glargine plus premeal insulin aspart) or continuous subcutaneous infusion **co-interventions:** nearly half the patients were using a statin; one third on anti-hypertensive therapy	**total number:** 30 (10/10) (10 in ramipril + ins arm) **mean age:** overall 46 yrs **gender:** overall ∼60% female **BMI:** overall ∼31–33 kg/m^2^ **ethnicity:** Mexican-American **diabetes duration:** overall 6.2 to 8.4 yrs **previous medication:** use of oral antidiabetic medications similar between groups	36 weeks	**primary:** vascular analyses **other:** HbA1c, hypoglycaemia: total daily dose, weight change, adverse events, vascular studies, lipid parameters
*Mattoo 2005* Worldwide [18]	effect of pioglitazone plus insulin versus placebo plus insulin on glycaemic control, serum lipid profile, and selected cardiovascular risk factors in patients with type 2 diabetes inadequately controlled with insulin therapy alone	**1) PIO + ins:** 30 mg pioglitazone plus insulin **2) P + ins:** identical placebo plus insulin **both:** insulin dose adjusted on basis of self- monitored blood glucose levels **co-interventions:** patients allowed other medication except another oral antidiabetic agent, systemic glucocorticoid therapy, or nicotinic acid (>500 mg/d).	**total number:** 289 (142/147) **mean age:** 58.8/58.9 yrs **gender (% female):** 56.3/57.1 **BMI:** 32.5/31.8 kg/m^2^ **ethnicity:** % White: 96.5/96.6 **diabetes duration:** 163.4/160.9 months **previous medication:** 149 patients previously on oral agents (MET n = 109, SU n = 19, MET + SU n = 17, other n = 4)	6 months	**primary:** HbA1c change **other:** hypoglycaemia, total daily dose, weight change, adverse events, lipid parameters
*Raz 2005* Worldwide [19] *Rosenstock 2002 (pioglitazone 014 study group)* USA[20]	efficacy and safety of biphasic insulin aspart 30/70 (BIAsp 30) plus pioglitazone versus glibenclamide plus pioglitazone and BIAsp 30 monotherapy in type 2 diabetes	**1) PIO + ins:** 30 mg pioglitazone once daily after breakfast plus biphasic insulin aspart 30/70 (BIAsp 30). BIAsp 30 initiated at a dose of 0.2 U/kg/day. **2) ins mono:** BIAsp 30 initiated at a dose of 0.3 U/kg/day **(PIO + glibenclamide** arm not considered here) **both:** biphasic insulin aspart 30/70 (BIAsp 30) **co-interventions:** no other insulin sensitizers; no manipulation of lipid lowering regimens	**total number:** 283 (93/ 97) **mean age:** 56.7/55.2 yrs **gender (% female);** 47/35 **BMI:** 29.4/29.5 kg/m^2^; **ethnicity:** NR **diabetes duration:** 9.2/10.0 yrs **previous medication:** % of patients taking other oral agents with SU; none: 14.0/13.4, acarbose: 9.7/12.4, meglitinides: 3.2/1/0, metformin: 83.9/80.4, thiazolidinediones: 7.5/4.1	18 weeks	**primary:** HbA1c **other:** hypoglycaemia, glycaemic excursions, total daily dose, weight adverse events, lipid profiles
	effect of two doses of pioglitazone (15 or 30 mg) in combination with a stable insulin regimen to improve glycaemic control in patients whose type 2 diabetes is poorly controlled on insulin therapy	**1) PIO15 + ins:** 15 mg pioglitazone plus usual insulin regimen **2)PIO30 + ins:** 30 mg pioglitazone plus usual insulin regimen **3) P + ins:** placebo plus usual insulin regimen **both:** insulin dose could be decreased in response to hypoglycaemia **co-interventions:** lipid-lowering medications allowed.	**total number:** 566 (191/188/187) **mean age:** 56.9/57.5/56.7 yrs **gender (% female):** 53.9/49.5/54.5 **BMI:** 33.2/34.3/33.2 kg/m^2^ **ethnicity (% Caucasian):** 74.9/73.4/71.1 **diabetes duration:** NR **previous medication:** 88% insulin monotherapy; 12% combination with oral agents; 134 patients receiving serum lipid reducing agent	16 weeks	**primary:** unclear presumably HbA1c **other:** hypoglycaemia, total daily dose, weight change, adverse events, serum lipid measurements
*Scheen 2006*19 European countries[21] part of PROactive trial (investigating only patients concomitantly treated with insulin) abstract only	effects of pioglitazone on the secondary prevention of macrovascular events in type 2 diabees	**1) PIO + ins:** pioglitazone plus previous treatment; forced titration phase in the first two months of treatment with stepwise increase of pioglitazone dose from 15 mg to 30 mg and then up to 45 mg **2) P + ins:** placebo plus previous treatment both: investigators encouraged to maintain glycaemia at <6.5% **co-interventions:** proportion of concomitant oral therapy (%): MET alone 47/52; SU alone 16/16; MET+SU 10/11	**total number:** 1760 (864/896) **age:** NR for subgroup on insulin **gender:** NR for subgroup on insulin **BMI:** NR for subgroup on insulin **ethnicity:** NR for subgroup on insulin **diabetes duration:** NR for subgroup on insulin **previous medication:** insulin + MET monotherapy in 53%, SU monotherapy in 24%, MET+SU 12% **subgroups:** abstract reports subgroup of larger trial where about one third of patients received concomitant insulin therapy	34.5 months (mean)	**primary:** (of PROactive trial) composite endpoint: any of all-cause mortality, non-fatal myocardial infarction, acute coronary syndrome, cardiac intervention, stroke, major leg amputation, bypass surgery; or revascularisation in leg **other:** HbA1c, hypoglycaemia, total daily dose, adverse events
*Shah 2007* USA[22] abstract only	effects of a pioglitazone and insulin combination versus insulin therapy alone on body fat distribution	**1) PIO + ins:** pioglitazone (30 mg titrated to 45 mg) and insulin **2) P + ins:** placebo and insulin **co-interventions:** NR	**total number:** 25 (12/13) **mean age:** NR **gender:** NR **BMI:** overall 36.5 kg/m^2^ **ethnicity:** NR **diabetes duration:** NR **previous medication:** NR	12 to 16 weeks	**primary:** body fat distribution **other:** HbA1c, weight change, subcutaneous adipose tissue, visceral adipose tissue (abdominal CT scans)

PIO + ins = pioglitazone plus insulin; P + ins = placebo plus insulin; NR = not reported; MET = metformin. SU = sulphonylurea.

#### Design

Seven trials were randomised double-blind placebo-controlled trials[15]–[18], [20]–[22], while one trial was a randomised open label trial[19]. The studies had different emphases: Asnani 2006 and Fernandez 2008 focussed on vascular reactivity; Berhanu 2007 focussed on reduction of insulin dosage; Mattoo 2005 focussed on glycaemic control, lipids and cardiovascular risk factors; Raz 2005 and Rosenstock 2002 focussed on glycaemic control; Scheen 2006 focussed on secondary prevention of macrovascular events; and Shah 2007 focussed on body fat distribution. Trial duration ranged between 12 weeks and 34.5 months. Five trials were from the USA[15]–[17], [20], [22], one included centres from a range of European countries[21], and two included centres worldwide[18], [19].

#### Participants

The trials included between 20 and 1760 participants, with between 10 and 896 participants in each comparison group. The total number of patients assessed was 3092. All studies included participants with previously inadequate glucose control (with different definitions, not reported for Shah 2007). Inclusion criteria with respect to previous treatment varied substantially. Only five trials[15], [17], [18], [20], [22] required previous insulin treatment. Three trials[15], [18], [20] required previous insulin therapy with or without oral antidiabetic agents (where reported, previous insulin monotherapy ranged between 48 and 88%). The trial by Fernandez 2008 required previous insulin combination therapy[17], and the trials by Shah 2007 included only insulin-treated obese patients[22]. Of the remaining trials, the trial by Berhanu 2007[16] required previous combination therapy with or without insulin, and in this trial between 90 and 93% of patients had been on sulphonylurea plus metformin therapy without insulin. The study by Raz 2005[19] required previous therapy with sulphonylurea (alone or as oral combination therapy) and over 80% of patients in that trial had been on sulphonylurea plus metformin previously. The study by Scheen 2006[21] included patients previously on diet alone, oral agents, or insulin plus an oral agent, and in that trial, over half the patients (53%) had been on sulphonylurea plus insulin, and the second largest group had been on sulphonylurea monotherapy (24%). Where reported, mean age of participants was between 46 to 59 years, the comparison groups included between 35 and 60% of women, mean BMI was between 29 and 37 kg/m^2^, and diabetes duration was between 6 and 14 years. The trial by Berhanu 2007[16] included between 50 and 59% of Hispanic participants, and the study by Fernandez 2008 included only Mexican-American participants[17].

#### Interventions

The trials used pioglitazone doses up to 45 mg/day. Four trials used titration schemes for pioglitazone (up to 45 mg/day, usually starting at 15 mg/day)[16], [17], [21], [22]. Three trials used fixed doses of 30 mg/day[15], [18], [19]. Rosenstock 2002 compared two pioglitazone doses, 15 and 30 mg/day[20].

As concerns the insulin therapy, Asnani 2006, Rosenstock 2002 and Scheen 2006 only specified that insulin therapy was continued as before. Rosenstock 2002 used a single blind insulin monotherapy lead-in period. Berhanu 2007 used a four week titration period for insulin (Humalog, Humulin 70/30 or Humulin N) and defined a target fasting plasma glucose of less than 140 mg/dL while avoiding hypoglycaemia. In the study by Fernandez 2008, patients could choose between multiple daily injections (basal-bolus therapy using combination of insulin glargine at bedtime plus premeal insulin aspart) or continuous subcutaneous infusions (basal infusion and premeal boluses of insulin aspart) and defined targets for blood glucose values (fasting and pre-meal capillary blood glucose 80–120 mg/dL, 2-h post-meal glucose <160 mg/dL, bedtime glucose <140 mg/dL). Mattoo 2005 used a three month insulin intensification period before randomisation; the insulin dose was reduced by 10% at randomisation to avoid hypoglycaemia and adjusted thereafter based on self-monitored blood glucose levels. Raz 2005 used biphasic insulin aspart 30/70. In the study by Scheen 2006, concomitant therapy with metformin was used by 47 to 52%, sulphonylurea alone by 16%, and metformin plus sulphonylurea by 10 to 11%. Shah 2007 did not give details of the insulin therapy.

Various studies specified co-interventions. Asnani 2006 allowed stable lipid lowering therapy with statins and anti-hypertensive therapy (including ACE inhibitors in all patients). In the study by Berhanu 2007 statins and metformin where continued as before. Fernandez 2008 changed all patients previously on ACE inhibitors or angiotensin II receptor blockers for blood pressure control to alpha-methyl dopa. Fernandez 2005 and Rosenstock 2002 allowed lipid lowering therapy as used before the study.

#### Outcomes

The trials used a variety of primary endpoints. HbA1c was the primary endpoint in the studies by Mattoo 2005, Raz 2005 and Rosenstock 2002. The primary endpoint in the study by Asnani 2006 was flow-mediated dilatation, in the study by Berhanu 2007 it was change in insulin dosage, Fernandez 2008 used vascular analyses as primary endpoint, the primary endpoint in the study by Scheen 2006 was a composite macrovascular endpoint, and in the study by Shah 2007 it was body fat distribution. All studies reported on end of study HbA1c values, six studies reported on hypoglycaemia[16]–[21], one study reported on glycaemic excursions[19], six studies reported on total daily dose[16]–[21], six studies reported on weight change[16]–[20], [22], five studies reported on adverse events[16], [18]–[21], six studies reported on lipid parameters[15]–[20], while none of the studies reported on rates of diabetic secondary complications or health-related quality of life.

### Study quality

Details of the quality of included trials are shown in Table 2.

**Table 2:** Quality of included trials.

Study	Method of randomisation	Allocation concealment	Blinding	Intention to treat data analysis	Percentage who completed trial	Power calculation	Similarity of groups at baseline	Sponsorship/author affiliation
Asnani 2006	carried out by research pharmacist using predetermined randomisation code	yes	double-blind	not reported	**PIO + ins:** 80% **P + ins:** 80%	yes (on flow-mediated dilatation)	HbA1c higher at baseline for pio+INS group (10% versus 8.7%) but not statistically significant	Takeda, NIH
Berhanu 2007	computer-generated schedule	yes	double-blind	yes	**PIO + ins:** 87.3% **P + ins:** 91.1%	yes (on mean change in insulin dose)	insulin group had significantly higher BMI (31.8 versus 30.7 kg/m^2^) and longer diabetes duration (8.5 versus 7.7 years)	Takeda Global R&D Centre
Fernandez 2008	not reported	not reported	double-blind	not reported	unclear – all?	yes (on vascular parameters)	yes	American Diabetes Association, Takeda Pharmaceuticals
Mattoo 2005	central randomisation table administered by an automated interactive voice system	yes	double-blind	yes	**PIO + ins:** 90% **P + ins:** 92%	yes (on change in HbA1c)	yes	Eli Lilly, Takeda Europe
Raz 2005	unclear (“assignment of lowest available patient number”)	not reported	no	yes	**PIO + ins:** 78% **ins mono:** 77%	yes (on change in HbA1c	yes	Novo Nordisk
Rosenstock 2002	not reported	not reported	double-blind	yes	**PIO15 + ins:** 84% **PIO30 + ins:** 91% **P + ins:** 88%	not reported	yes	Takeda Pharmaceuticals
Scheen 2006	central interactive voice-response system	not reported	double-blind	yes	not reported	yes	not reported	Takeda Europe, Eli Lilly
Shah 2007	not reported	not reported	double-blind	not reported	not reported	not reported – small numbers, probably underpowered	not reported	not reported

For four[15], [16], [18], [19] of the eight trials, randomisation was adequate, while for the remaining four trials the randomisation procedure was not reported or unclear. Three trials[15], [16], [18] had adequate allocation concealment, while the rest of the trials did not report on allocation concealment. All but one trial[19] were described as double-blind. Five trials used intention-to-treat analysis[16], [18]–[21]. Five trials reported on follow-up rates[15], [16], [18]–[20] and in those trials, between 77 and 92% of participants completed the trial, without any significant differences between comparison groups. Six of the eight trials reported that they had carried out a power calculation[15]–[18], [20], [21]. Six trials were reported in full and two were only available as meeting abstracts. The two trials reported as abstracts[21], [22] did not report relevant baseline characteristics, five trials reported that their comparison groups were similar at baseline[15], [17]–[20], while Berhanu 2007[16] stated that participants in the placebo group had a slightly higher BMI at baseline and longer diabetes duration, but it was unclear whether these differences were significant. All but one trial[22] reported on sources of funding and all funding included industry funding.

### Data synthesis

Results of the individual trials are shown in Table 3.

**Table 3:** Results of included trials.

Study	Outcome	Baseline	End of study	Change from baseline/difference between groups	p value (between groups)
**HbA1c**					
Asnani 2006	HbA1c (%)	**PIO + ins:** 10.0 SD2.3% **P + ins:** 8.7 SD2.3%	**PIO + ins:** 8.4 SD2.0% **P + ins:** 8.6 SD1.4%		p not reported (p<0.05 for pio before and after)
Berhanu 2007	HbA1c (%)	**PIO + ins:** 8.4 SE0.13% **P + ins:** 8.6 SE0.13%	**PIO + ins:** 6.81% **P + ins:** 7.23%	**PIO + ins:** −1.6 SE0.11% **P + ins:** −1.4 SE0.11%	p = NS
Fernandez 2008	HbA1c (%)	**PIO + ins:** 9.0 SD0.7% **P + ins:** 9.2 SD0.4%	**PIO + ins:** 6.9 SD0.3% **P + ins:** 7.2 SD0.1%		
Mattoo 2005	HbA1c (%)	**PIO + ins:** 8.85 SE0.11% **P + ins:** 8.79 SE0.1%	**PIO + ins:** 8.11 SE0.09% **P + ins:** 8.66 SE0.08%	**PIO + ins:** −0. 69 SE0.09% **P + ins:** −0.13% difference between groups −0.55 SE0.1%	p<0.002
	percentage attaining HbA1c <7.0%		**PIO + ins:** 18% **P + ins:** 6.9%		
	HbA1c subgroups: patients using ≤2 or ≥3 insulin injections				no significant difference
	HbA1c subgroups: previous use of oral antidiabetic agents			**previous use of oral agents:** **PIO + ins:** −0.90 SE0.14%; **P + ins:** −0.11 SE0.13% **no previous use of oral agents:** **PIO + ins:** −0.65 SE0.11%; **P + ins:** −0.2 SE0.12%	no significant difference for subgroups
Raz 2005	HbA1c (%)	**PIO + ins:** 9.6 SD1.3% **ins mono:** 9.5 SD1.3%	**PIO + ins:** 8.4 SD1.2% **ins mono:** 9.0 SD1.3%		p = 0.008
Rosenstock 2002	HbA1c (%)	**PIO15 + ins:** 9.75 SE0.1% **PIO30 + ins:** 9.84 SE0.1% **P + ins:** 9.75 SE0.1%		**PIO15 + ins:** −0.99 SE0.08% **PIO30 + ins:** −1.26 SE0.08% **P + ins:** −0.26 SE0.08%	p<0.01 pioglitazone versus placebo
Shah 2007	HbA1c (%)	**PIO + ins:** 7.6% **P + ins:** 7.8%	**PIO + ins:** 7.1% **P + ins:** 7.2%		p not reported, presumably non-significant
Scheen 2006	HbA1c (%)	**PIO + ins:** 8.4% **P + ins:** 8.5%	**PIO + ins:** 7.47% **P + ins:** 8.05%	**PIO + ins:** −0.93% **P + ins:** −0.45%	p<0.0001
**hypoglycaemia**					
Berhanu 2007	patients with hypoglycaemic events		**PIO + ins:** 46% (91% mild) **P + ins:** 31% (66% mild)		p<0.005
	severe hypoglycaemia (episodes)		**PIO + ins:** n = 0 **P + ins:** n = 4		p not reported
Fernandez 2008	patients with hypoglycaemic episodes		**PIO + ins:** n = 4 **P + ins:** n = 6		
Mattoo 2005	patients with subjective hypoglycaemic episodes		**PIO + ins:** 63.4% **P + ins:** 51.0%		p<0.05
	clinical hypoglycaemic episodes (blood glucose <2.8 mmol/L)				no significant difference
Raz 2005	major hypoglycaemic episodes		none		
	minor hypoglycaemic episodes (% patients)		**PIO + ins:** 12% **ins mono:** 15%		p not reported
	minor hypoglycaemic episodes (episodes)		**PIO + ins:** 15 **ins mono:** 47		p not reported
	symptoms only (% patients)		**PIO + ins:** 34% **ins mono:** 40%		p not reported
	symptoms only (episodes)		**PIO + ins:** 115 **ins mono:** 171		p not reported
	incidence (per patient-week for all episodes)		**PIO + ins:** 0.083 **ins mono:** 0.132		p<0.05
	nocturnal hypoglycaemia (episodes)		**PIO + ins:** 0 **ins mono:** 8		p not reported
Rosenstock 2002	hypoglycaemia		**PIO15 + ins:** 8% **PIO30 + ins:** 15% **P + ins:** 5% (all considered mild to moderate)		
Scheen 2006	hypoglycaemia (not specified further)		**PIO + ins:** 41% **P + ins:** 29%		p<0.0001
**glycaemic excursions**					
Raz 2005					measurements before dinner, 90 mins after dinner, and at bedtime significantly lower in PIO + ins group than in ins monotherapy group
**total daily dose**					
Berhanu 2007	daily insulin dose	**PIO + ins:** 55.8 SE2.95 units **P + ins:** 57.7 SE2.95 units		**PIO + ins:** −12.0 SE1.84 units **P + ins:** +0.8 SE1.84 units adjusted mean difference between groups −12.7 units (95% CI: −17.5, −8.0)	p<0.001
Fernandez 2008	daily insulin dose	**all groups:** ∼1.2 U/kg/day	**PIO + ins:** 1.0 U/kg/day **P + ins:** ∼1.2 U/kg/day		p not reported
Mattoo 2005	daily insulin dose	**PIO + ins:** 0.96 SE0.03 U/kg/day **P + ins:** 0.92 SE0.03 U/kg/day	**PIO + ins:** 0.76 SE0.02 U/kg/day **P + ins:** 0.94 SE0.02 U/kg/day	difference between groups −0.18 SE0.02 U/kg/day	p<0.002
Raz 2005	daily insulin dose	**PIO + ins:** 0.2 U/kg/day **ins mono:** 0.3 U/kg/day	**PIO + ins:** 0.5 U/kg/day **ins mono:** 0.7 U/kg/day	**PIO + ins:** +0.3 U/kg/day **ins mono:** +0.4 U/kg/day	p = 0.002
Rosenstock 2002	daily insulin dose	**PIO15 + ins:** 70.2 SE34.0 U/day **PIO30 + ins:** 72.3 SE38.5 U/day **P + ins:** 70.7 SE33.5 U/day	**PIO15 + ins:** 67.3 SE33.5 U/day **PIO30 + ins:** 64.2 SE32.7 U/day **P + ins:** 70.1 SE33.9 U/day		p not reported
Scheen 2006	daily insulin dose	**PIO + ins:** 47 U/day **P + ins:** 47 U/day	**PIO + ins:** 42 U/day **P + ins:** 55 U/day		p<0.0001; at final visit, insulin discontinued in 9% of pioglitazone group and 2% of placebo group (p<0.0001)
**weight change**					
Berhanu 2007	weight (kg)			**PIO + ins:** +4.39 kg **P + ins:** +2.42 kg	p not reported
	patients reporting weight gain			**PIO + ins:** n = 10 **P + ins:** n = 3	p not reported
Fernandez 2008	weight (kg)			**PIO + ins:** +4.4 kg **P + ins:** +1.7 kg	p not reported
Mattoo 2005	weight (kg)			**PIO + ins:** +4.05 SE4.03 kg **P + ins:** +0.20 SE2.92 kg	p not reported
Raz 2005	weight (kg)			**PIO + ins:** +4.0 kg **ins mono:** +2.2 kg	p not reported
	patients experiencing weight gain (%)			**PIO + ins:** 8% **ins mono:** 2%	p not reported
Rosenstock 2002	weight (kg)	**PIO15 + ins:** 95.4 SE17.6 kg **PIO30 + ins:** 98.7 SE17.7 kg **P + ins:** 95.4 SE17.0 kg		**PIO15 + ins:** +2.3 kg **PIO30 + ins:** +3.7 kg **P + ins:** −0.04 kg	p not reported; weight gain related to decreases in HbA1c, p = 0.002
Shah 2007	weight (kg)	**PIO + ins:** 107.1 kg **P + ins:** 108.7 kg	**PIO + ins:** 112.0 kg **P + ins:** 110.1 kg		p not reported, presumably non-significant
**lipid parameters**					
Berhanu 2007	total cholesterol (mg/dL)	**PIO + ins:** 178 SE3.53 mg/dL **P + ins:** 183 SE3.6 mg/dL		**PIO + ins:** +5.7 SE2.75 mg/dL **P + ins:** +4.7 SE2.78 mg/dL	p = NS
	HDL cholesterol (mg/dL)	**PIO + ins:** 44.6 SE1.3 mg/dL **P + ins:** 42 SD1.3 mg/dL		**PIO + ins:** +4.3 SE0.75 mg/dL **P + ins:** −0.2 SE0.77 mg/dL	p<0.001
	LDL cholesterol (mg/dL)	**PIO + ins:** 107 SE3.1 mg/dL **P + ins:** 111 SE3.2 mg/dL		**PIO + ins:** +4.0 SE2.37 mg/dL **P + ins:** +0.9 SE2.37 mg/dL	p = NS
	triglycerides (mg/dL)	**PIO + ins:** 123 SE7.5 mg/dL **P + ins:** 141 SE7.6 mg/dL		**PIO + ins:** −0.2 SE9.80 mg/dL **P + ins:** +43.7 SE9.96 mg/dL	p<0.001
Fernandez 2008	total cholesterol (mg/dL)	**PIO + ins:** 176 SD9 mg/dL **P + ins:** 195 SD9 mg/dL	**PIO + ins:** 175 SD16 mg/dL **P + ins:** 180 SD8 mg/dL		p = NS
	LDL cholesterol (mg/dL)	**PIO + ins:** 107 SD7 mg/dL **P + ins:** 121 SD8 mg/dL	**PIO + ins:** 105 SD12 mg/dL **P + ins:** 115 SD7 mg/dL		p = NS
	HDL cholesterol (mg/dL)	**PIO + ins:** 45 SD3 mg/dL **P + ins:** 49 SD4 mg/dL	**PIO + ins:** 51 SD3 mg/dL **P + ins:** 46 SD3 mg/dL		p<0.05 pioglitazone versus baseline
	VLDL cholesterol (mg/dL)	**PIO + ins:** 109 SD16 mg/dL **P + ins:** 113 SD24 mg/dL	**PIO + ins:** 88 SD15 mg/dL **P + ins:** 93 SD19 mg/dL		
	triglycerides (mg/dL)	**PIO + ins:** 148 SD17 mg/dL **P + ins:** 146 SD15 mg/dL	**PIO + ins:** 123 SD11 mg/dL **P + ins:** 132 SD18 mg/dL		
Mattoo 2005	HDL cholesterol (mmol/L)	**PIO + ins:** 1.23 SE0.03 mmol/L **P + ins:** 1.24 SE0.03 mmol/L	**PIO + ins:** 1.35 SE0.02 mmol/L **P + ins:** 1.21 SE0.02 mmol/L	difference between groups 0.13 SE0.03 mmol/L	p<0.002
	LDL cholesterol (mmol/L)	**PIO + ins:** 3.20 SE0.09 mmol/L **P + ins:** 3.18 SE0.08 mmol/L	**PIO + ins:** 3.18 SE0.06 mmol/L **P + ins:** 3.10 SE0.06 mmol/L		p = NS
Raz 2005	triglycerides (mg/dL)		**PIO + ins:** 149 SD88 mg/dL **ins mono:** 158 SD88 mg/dL		p = NS
	total cholesterol (mg/dL)		**PIO + ins:** 212 mg/dL **ins mono:** 204 mg/dL		p = NS
	HDL cholesterol (mg/L)			difference between PIO + ins versus ins mono +4 SD1 mg/dL	p<0.01
	LDL cholesterol (mg/L)			no data shown	p = NS
Rosenstock 2002	triglycerides (mmol/L)	**PIO15 + ins:** 2.61 SE0.2 mmol/L **PIO30 + ins:** 2.96 SE0.2 mmol/L **P + ins:** 2.74 SE0.2 mmol/L		LS % mean change from baseline **PIO15 + ins:** +5.35 SE6.56% **PIO30 + ins:** −10.35 SE6.54% **P + ins:** +13.30 SE6.63%	p<0.05 PIO30 versus placebo
	HDL cholesterol (mg/dL)	**PIO15 + ins:** 43.42 SE0.95 mg/dL **PIO30 + ins:** 42.71 SE0.94 mg/dL **P + ins:** 42.66 SE0.96 mg/dL		LS % mean change from baseline **PIO15 + ins:** +7.07 SE1.58% **PIO30 + ins:** +9.13 SE1.57% **P + ins:** −0.21 SE1.59%	p<0.05 PIO30 versus placebo
	total cholesterol (mg/dL)	**PIO15 + ins:** 213.08 SE3.57 mg/dL **PIO30 + ins:** 207.32 SE3.53 mg/dL **P + ins:** 214.03 SE3.58 mg/dL		LS % mean change from baseline **PIO15 + ins:** +1.40 SE1.06% **PIO30 + ins:** +0.40 SE1.05% **P + ins:** −0.66 SE1.07%	p = NS
	LDL cholesterol (mg/dL)	**PIO15 + ins:** 127.33 SE3.07 mg/dL **PIO30 + ins:** 121.69 SE3.06 mg/dL **P + ins:** 130.95 SE3.05 mg/dL		LS % mean change from baseline **PIO15 + ins:** +2.83 SE1.80% **PIO30 + ins:** +5.05 SE1.71% **P + ins:** −1.41 SE1.74%	p = NS
**adverse events**					
Berhanu 2007	oedema			**PIO + ins:** n = 10 **P + ins:** n = 5(all mild to moderate)	p not reported
	serious adverse events			**PIO + ins:** n = 4 **P + ins:** n = 2(none considered to be related to study medication)	p not reported
Fernandez 2008	mild peripheral oedema			**PIO + ins:** n = 3 **P + ins:** n = 0	p not reported
Mattoo 2005	withdrawal due to adverse events			**PIO + ins:** n = 7 **P + ins:** n = 3	p not reported
	oedema			**PIO + ins:** n = 20 (10 classified as mild) **P + ins:** n = 5 (3 classified as mild)	p not reported
Raz 2005	withdrawal due to adverse events			**PIO + ins:** n = 1 **ins mono:** n = 2	p not reported
	patients with product-related adverse events			**PIO + ins:** 28% **ins mono:** 20%	p not reported
	peripheral oedema			**PIO + ins**: 6% **ins mono:** 0	p not reported
	serious adverse events			**PIO + ins:** n = 0 **ins mono:** n = 2 (none considered to be related to study medication)	
Rosenstock 2002	withdrawal due to adverse events			**PIO15 + ins:** 1.6% **PIO30 + ins:** 2.6% **P + ins:** 3.2%	p not reported
	oedema			**PIO15 + ins:** 12.6% **PIO30 + ins:** 17.6% **P + ins:** 7.0%	p not reported
Scheen 2006	oedema			**PIO + ins:** 31% **P + ins:** 18%	p<0.0001
**cardiac adverse events**					
Berhanu 2006				insulin only patients had a higher incidence of cardiac events (10.7% versus 5.5%), the majority of which were ECG abnormalities; one patient each with myocardial infarction and cardiac hypertrophy in the insulin only group; one patient with coronary artery disease in the pioglitazone group; no deaths	
Raz 2005				2 cases of myocardial infarction in insulin monotherapy group (not considered to be treatment-related)	
Rosenstock 2002				rate of cardiovascular adverse events 7.9% pioglitazone, 7.0% insulin only, no significant difference; congestive heart failure in 2 patients receiving 15 mg/day pioglitazone and in 2 patients receiving 30 mg/day pioglitazone, all in patients with history of cardiovascular disease and none considered to be drug-related	
**HR QoL**	not reported				

HR QoL = health-related quality of life; SD = standard deviation; SE = standard error

**Figure 2:**
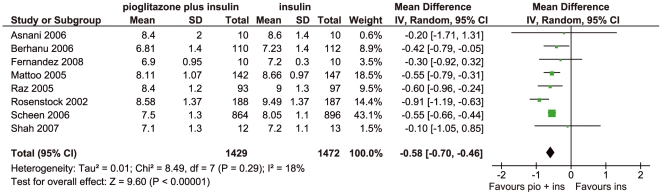
Forest plot of HbA1c results; SD = standard deviation, IV = inverse variance method, pio = pioglitazone, ins = insulin.

*HbA1c.* All studies reported HbA1c values and could be included in the meta-analysis (Figure 2). Baseline HbA1c values were between 7.6 and 10% in the pioglitazone plus insulin groups and between 7.8 and 9.8% in the insulin without pioglitazone groups. End-of-study HbA1c values were significantly lower in the groups taking pioglitazone plus insulin than in the groups taking insulin without pioglitazone (weighted mean difference −0.58%, 95% CI: −0.70, −0.46, p<0.00001). There was no significant heterogeneity. In the study by Mattoo 2005, 18% of patients on pioglitazone plus insulin and 6.9% of patients on insulin without pioglitazone attained HbA1c values of below 7.0%. There was no significant difference between patients using two or fewer daily injections and patients using three or more daily injections. Similarly, there was no significant difference between patients who had previously been on oral antidiabetic agents and those who had not been on oral agents. In the study by Rosenstock 2002, no significant difference in HbA1c was reported for the group using 15 mg/day of pioglitazone and the group using 30 mg/day. There was no significant difference in HbA1c results when comparing studies in which the insulin regimen was unchanged from before the study[15], [20]–[22] and studies using titrated insulin regimens according to a predefined study protocol[16]–[19] (HbA1c difference −0.63%, 95% CI: −0.93, −0.34, with insulin as usual, compared to −0.52%, 95% CI: −0.68, −0.35, with insulin as per study protocol, p = 0.44).

#### Hypoglycaemia

**Figure 3:**
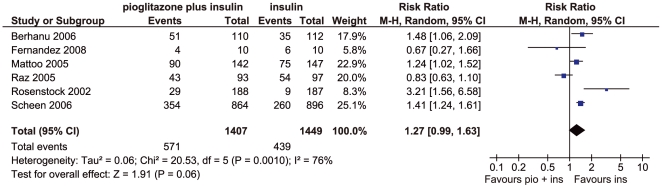
Forest plot of frequency of hypoglycaemia; M-H = Mantel-Haenszel, pio = pioglitazone, ins = insulin.

Six studies reported on hypoglycaemia outcomes and could be summarised in a meta-analysis (Figure 3). There were marginally more patients with hypoglycaemic episodes in the pioglitazone plus insulin groups than with insulin without pioglitazone (relative risk 1.27, 95% CI: 0.99, 1.63, p = 0.06). The results showed significant heterogeneity (p = 0.001). The study by Raz 2005 which used biphasic insulin aspart 30 (BIAsp 30) rather than other insulin regimens contributed most to the heterogeneity. There is evidence to suggest that BIAsp 30 is associated with a reduced rate of nocturnal and major episodes of hypoglycaemia compared to other types of insulin[23]. After eliminating this study from the analysis, there remained moderate heterogeneity (I^2^ = 57%, p = 0.05) and there was significantly more hypoglycaemia in the pioglitazone plus insulin groups (relative risk 1.40, 95% CI: 1.14, 1.73, p = 0.002). There were no significant differences in intervention and control groups for hypoglycaemia either for studies in which the insulin regimen was unchanged from before the study[15], [20]–[22] or for studies using titrated insulin regimens according to a predefined study protocol[16]–[19]. Details regarding hypoglycaemic episodes are shown in Table 4. Severe hypoglycaemic events were rarely seen in the studies.

**Table 4:** Details of hypoglycaemic episodes.

Study	Definition of hypoglycaemia	Classification of hypoglycaemic episodes
Asnani 2006	hypoglycaemia not reported	hypoglycaemia not reported
Berhanu 2006	self-monitored blood glucose <3.3 mmol/L or laboratory value <3.9 mmol/L, more than two simultaneous hypoglycaemia symptoms relieved by oral glucose-containing substance, or resulting in needing assistance for simple tasks	more hypoglycaemic events in the pioglitazone group, but almost all (91%) rated as mild compared to 66% in the insulin only group; 0 severe hypoglycaemic events versus 4 in the insulin only group
Fernandez 2008	symptomatic hypoglycaemia requiring glucose ingestion	6 patients in insulin only group and 4 patients in pioglitazone plus insulin group with hypoglycaemic episodes as defined; 33 hypoglycaemic episodes (0.32 patients per year)
Mattoo 2005	1) subjective symptoms only, 2) subjective symptoms with a self-monitored blood glucose level ≥2.8 mmol/L, 3) subjective symptoms with a self-monitored blood glucose level <2.8 mmol/L, and 4) self-monitored blood glucose <2.8 mmol/L without symptoms; severe: patient either had blood glucose <2.8 mmol/L or promptly recovered after oral carbohydrate, glucagon, or intravenous glucose, but required the assistance of another person for recovery, non-severe: patient did not require assistance of another person for recovery, regardless of blood glucose level	no difference between groups in rate of hypoglycaemic incidents or number of clinical hypoglycaemic episodes (blood glucose <2.8 mmol/L); 63.4% of patients with subjective hypoglycaemic episodes in pioglitazone plus insulin group versus 51.0% for insulin only (p<0.05) but no difference for other types of hypoglycaemia
Raz 2005	major: unable to self-treat, blood glucose <2.8 mmol/L, or symptoms remitted after administration of intravenous glucose or intramuscular glucagon or after food intake; minor: blood glucose <2.8 mmol/L and patient handled the event without assistance from others; symptomatic: hypoglycaemic symptoms present but not confirmed with blood glucose measurement, assistance from others not required	no major hypoglycaemic episodes; 56% of patients in insulin only group had hypoglycaemic episodes (72% symptoms only, 28% minor), 8 events of nocturnal hypoglycaemia; 46% of patients in the pioglitazone group had hypoglycaemic events (74% symptomatic, 26% minor), no nocturnal hypoglycaemia; p-value not reported
Rosenstock 2002	fasting plasma glucose ≤5.6 mmol/L on two occasions or symptoms of hypoglycaemia not explained by other conditions	all considered mild or moderate; most self-treated with caloric intake; all reported while patients were at home
Scheen 2006	not reported	not reported
Shah 2007	hypoglycaemia not reported	hypoglycaemia not reported

#### Insulin dose

Six studies[16]–[21] reported insulin doses (as units per kg per day or as units per day). Only two studies reported standard deviations, so a meta-analysis could not be carried out reliably. Of the six studies, four found that the insulin plus pioglitazone groups used significantly less insulin than the insulin without pioglitazone groups (weighted mean difference −0.19 U/kg/day or −12.03 U/day). The remaining two studies did not report any p-values (but doses were also lower in the pioglitazone groups). Insulin dose ranged between 42 and 64 U/day or 0.5 to 1 U/kg/day in the pioglitazone groups and between 55 and 70 U/day or 0.7 to 1.2 U/kg/day in the groups taking no pioglitazone.

#### Weight change

Six studies reported weight change[16]–[20], [22]. However, only one of the studies reported a measure of variability, so a meta-analysis could not be carried out reliably. In most studies, patients in the insulin without pioglitazone groups gained less weight than patients in the insulin plus pioglitazone groups (mean difference 2.91 kg, range 3.85 to −3.50 kg), but no p-values were reported. Weight change ranged between +1.4 and +4.4 kg in the pioglitazone plus insulin groups and between −0.04 and +4.9 kg in the insulin only groups.

#### Lipid parameters

Four studies reported results for serum triglycerides [16], [17], [19], [20].

Of the four studies, only two[16], [20] found significantly reduced triglyceride values in the pioglitazone groups (reductions of between 0.44 and 0.70 mmol/L in the pioglitazone groups compared to insulin only).

Four studies reported on total serum cholesterol[16], [17], [19], [20]. None of the studies found any significant difference in total cholesterol between the pioglitazone plus insulin and the insulin without pioglitazone groups.

Four studies reported on HDL-cholesterol[16]–[18], [20], all finding significantly increased values in the pioglitazone groups. Overall, HDL cholesterol was increased by between 0.10 and 0.18 mmol/L in the pioglitazone groups compared to insulin only.

Four studies reported on LDL-cholesterol[16]–[18], [20], with none finding any significant difference between the pioglitazone plus insulin and the insulin without pioglitazone groups.

#### Adverse events

Where reported, there did not appear to be any significant difference in withdrawals due to adverse events between the pioglitazone plus insulin and the insulin without pioglitazone groups. Apart from weight gain and hypoglycaemia, the only adverse event reported as occurring more frequently with pioglitazone was (peripheral) oedema, which was generally classified as mild to moderate. However, p-values were generally not reported.

Only three trials reported on cardiovascular adverse events[16], [19], [20] (and most studies were probably underpowered for coming to reliable conclusions). In the trial by Berhanu 2006, insulin only patients had a higher incidence of cardiac events (10.7% versus 5.5%) but the majority were ECG abnormalities rather than patient oriented outcomes; there was one patient each with myocardial infarction and cardiac hypertrophy in the insulin only group, and one patient with coronary artery disease in the pioglitazone group; no deaths were observed. Raz 2005 reported two cases of myocardial infarction in the insulin monotherapy group which were not considered to be treatment-related. Rosenstock 2002 observed a rate of cardiovascular adverse events of 7.9% with pioglitazone plus insulin and of 7.0% with insulin only (no significant difference); congestive heart failure was seen in two patients receiving 15 mg/day pioglitazone and in two patients receiving 30 mg/day pioglitazone. All cases were in patients with a history of cardiovascular disease and none were considered to be drug-related.

#### Heterogeneity

We performed a range of subgroup analyses, these included: insulin therapy “as usual” versus insulin individually titrated for the study, pioglitazone increased/titrated up to 45 mg versus constant dose of 30 mg, abstract only versus full publication, baseline HbA1c <8.5 or 9% versus ≥8.5 or 9%, comparison group n<100 versus n>100, study duration <6 months versus ≥6 months, study quality 5 or 6 or more criteria fulfilled versus less than 5 or 6 criteria fulfilled. No significant differences in study results were seen for any of the subgroup analyses.

## Discussion

### Summary

Eight randomised controlled trials were identified comparing combinations of insulin and pioglitazone with insulin without pioglitazone regimens (two published as abstracts only). Compared to the insulin regimens, the pioglitazone plus insulin regimens reduced HbA1c by a mean of −0.58% (95% CI: −0.70, −0.46, p<0.00001). However, hypoglycaemic events were marginally increased with the pioglitazone regimens (relative risk 1.27, 95% CI: 0.99, 1.63, p = 0.06). Where reported, studies found reduced insulin doses in the pioglitazone groups. HDL-cholesterol was increased, but none of the other lipid parameters reported (triglycerides, total cholesterol, LDL-cholesterol) showed any systematic differences between the comparison groups. The studies tended to show increased weight (mean difference 2.91 kg) and more peripheral oedema with pioglitazone. No conclusions could be made regarding cardiovascular events.

This review adds to the current body of evidence in two ways. First, by demonstrating some added value of pioglitazone when added to insulin-containing regimens, and secondly by making the point that starting patients on insulin does not mean that all further intensifications need to occur by adding more insulin.

### Limitations

The main evidence gap relates to long-term safety. The studies included in this review were too short-term and too few in number to assess longer-term adverse effects. Recent debates on the TZDs have focused on cardiovascular events, following the review by Nissen and colleagues[24], which concluded that the risk of cardiovascular events was increased by rosiglitazone. A meta-analysis by Monami et al. (2008)[25] aimed to identify moderators of the effect of rosiglitazone on incidence of myocardial infarction and found that there was a significant correlation between the proportion of insulin-treated patients and rosiglitazone-associated risk of myocardial infarction (r = 0.42, p<0.05) (possibly related to the fact that both thiazolidinediones and insulin have an effect on fluid retention). Similar data are not available for pioglitazone.

A meta-analysis of the risk of cardiovascular events with pioglitazone was carried out by Lincoff et al. (2007)[26]. Based on 19 trials with 16,930 participants, they concluded that pioglitazone was associated with a reduced risk of death, myocardial infarction or stroke.

Another meta-analysis by Mannucci and colleagues[27] included 84 published and 10 unpublished trials of pioglitazone compared to placebo or active comparators, but excluded the PROactive trial. They reported a reduction of all-cause mortality with pioglitazone (OR 0.30; 95% CI: 0.14 to 0.63: p<0.05), but no significant effect on non-fatal coronary events.

Several new studies have asked why rosiglitazone should increase cardiovascular events but pioglitazone does not. Most have concluded that the likely reason is that while the two glitazones have the same effects on glycaemic control, and the same side-effects of fluid retention and heart failure, they have different effects on blood lipids. Berneis and colleagues[28] carried out a very small cross-over trial in 9 patients, giving them all 12 weeks on pioglitazone and 12 weeks on rosiglitazone. Total cholesterol increased more on rosiglitazone than on pioglitazone (p = 0.04), and triglycerides increased on rosiglitazone but decreased on pioglitazone (p = 0.004).

Chappuis and colleagues[29] also studied patients on both glitazones, this time with 17 patients having 12 weeks on each. The effects of HbA1c were similar, but triglyceride and cholesterol levels were lower with pioglitazone.

Deeg and colleagues[30] carried out a much larger comparison with 369 participants randomised to pioglitazone and 366 to rosiglitazone. After 12 weeks, pioglitazone had reduced LDL whereas rosiglitazone had increased it.

The other concern about the safety of the glitazones has been the fracture risk. Kahn and colleagues[31] in the “durability” study (ADOPT) reported that 9.3% of women on rosiglitazone had fractures compared to 5.1% on metformin and 3.5% on glibenclamide. The increases were in fractures of upper limb and foot, rather than in the classical osteoporosis-associated neck of femur and vertebrae. The fracture rates in men did not differ between treatment groups.

A case-control study by Meier and colleagues[32] using British general practice data from GPRD also found that use of glitazones was associated with increased fracture rates. No such increase was seen with other oral diabetes drugs.

A recent (non-systematic) review by Schwartz (2008)[33] summarised clinical data on bone loss associated with use of glitazones. This reiterates data from recent trials reporting increased fracture risk in women (but not men) and reports evidence from short term clinical trials in women that glitazones (both pioglitazone and rosiglitazone) caused more rapid bone loss. It also reports data from in vitro and rodent models suggesting that activation of the PPAR-gamma receptor can play a role in bone loss.

A letter to physicians issued by Takeda Pharmaceuticals (posted on the US Food and Drug Administration website in March 2007) reported an analysis of its clinical trials database on pioglitazone[34]. They compared the incidence of fractures in over 8100 patients treated with pioglitazone compared to over 7400 patients treated with a comparator. The fracture incidence calculated was 1.9 fractures per 100 patient years in women treated with pioglitazone and 1.1 fractures per 100 patient years in women treated with a comparator. The observed excess risk of fractures for women in this dataset on pioglitazone is therefore 0.8 fractures per 100 patient years of use. There was no increased risk of fractures identified in men.

The more drugs a patient has to take, the poorer the adherence. Donnan and colleagues from Dundee[35] found that even those on only one glucose lowering agent have poor adherence, with adequate adherence in only one in three. Adherence is better with a single daily dose[35]. Those taking other medications had poorer compliance than those on just a hypoglycaemic agent.

A systematic review of medication adherence in patients with poorly controlled diabetes by Odegard and colleagues[36] summarises the barriers to taking medicines, and the interventions which may help. Some of the studies are more relevant to the North American situation where people have to pay for drugs, but much of it is relevant to the UK. The review concurs with the work of Donnan and colleagues (mentioned above), that common barriers to adherence include complexity of regimen and number of doses.

The implication for the treatment of type 2 diabetes may be that both the number of drugs and the number of tablets or injections per day should be kept as low as possible[37].

### Conclusions

When added to insulin regimens, pioglitazone confers a small but clinically useful decrease in HbA1c of 0.58% in type 2 diabetes patients with previous inadequate glucose control. However it does so at the cost of increased hypoglycaemia and weight gain, with a risk of heart failure, and of fractures in women. The effect on adherence of adding another medication needs to be considered, as does the extra cost, though that is offset by the reduced insulin dose required.
